# Patients’ and relatives’ perspectives on the quality of end-of-Life care in advanced cancer: From the final months to bereavement

**DOI:** 10.1371/journal.pone.0342068

**Published:** 2026-02-09

**Authors:** Moyke A. J. Versluis, Yvette M. van der Linden, Lobke van Leeuwen-Snoeks, Marieke H. J. van den Beuken-van Everdingen, Mathijs P. Hendriks, Ben E. E. M. van den Borne, Dirkje Sommeijer, Jean-Paul A. van Basten, Annemieke van der Padt-Pruijsten, Alexander de Graeff, Jarmo C. B. Hunting, Evelien J. M. Kuip, Anne S. R. van Lindert, Magdolen Youssef-El Soud, Martine F. Thijs-Visser, Art Vreugdenhil, Hanneke W. M. van Laarhoven, Wouter K. de Jong, Caroline Mandigers, Lia van Zuylen, Jeroen kloover, Tineke J. Smilde, Lonneke V. van de Poll-Franse, Natasja J. H. Raijmakers

**Affiliations:** 1 Research and development, Netherlands Comprehensive Cancer Organisation (IKNL), Utrecht, the Netherlands; 2 Graduate school of social and behavioral sciences, Tilburg University, Tilburg, the Netherlands; 3 Centre of expertise in palliative Care, Leiden University Medical Centre, Leiden, the Netherlands; 4 Department of Radiotherapy, Leiden University Medical Centre, Leiden, the Netherlands; 5 Department of internal medicine, Diakonessenhuis, Utrecht, the Netherlands; 6 Centre of expertise in Palliative Care, Maastricht University Medical Centre (MUMC+), Maastricht, the Netherlands; 7 Department of Anesthesiology and Pain Management, Maastricht University Medical Centre+ (MUMC+), Maastricht, the Netherlands; 8 Department of Medical Oncology, Northwest Clinics, Alkmaar, the Netherlands; 9 Department of Pulmonology, Catharina Hospital, Eindhoven, the Netherlands; 10 Department of Internal Medicine, Flevoziekenhuis, Almere, the Netherlands; 11 Department of Urology, Canisius Wilhelmina Hospital, Nijmegen, the Netherlands; 12 Department of Internal Medicine, Maasstad Hospital, Rotterdam, the Netherlands; 13 Department of Internal Medicine, Spijkenisse MC, Spijkenisse, the Netherlands; 14 Department of Medical Oncology, University Medical Center Utrecht, Utrecht, the Netherlands; 15 Department of Medical Oncology, St. Antonius Hospital, Utrecht, the Netherlands; 16 Department of Medical Oncology, Radboud University Medical Center, Nijmegen, the Netherlands; 17 Department of Pulmonology, University Medical Center Utrecht, Utrecht, the Netherlands; 18 Department of lung oncology, Maxima Medical Centre, Eindhoven, the Netherlands; 19 Department of Internal Medicine, Ikazia Hospital, Rotterdam, the Netherlands; 20 Department of Medical Oncology, Maxima Medical Centre, Veldhoven, the Netherlands; 21 Cancer Center Amsterdam, Cancer Treatment and Quality of Life, Amsterdam, the Netherlands; 22 Department of Medical Oncology, Amsterdam University Medical Centre location AMC, Amsterdam, the Netherlands; 23 Department of Pulmonology, University Medical Centre Utrecht, Utrecht, the Netherlands; 24 Department of Internal Medicine, Canisius Wilhelmina Hospital, Nijmegen, the Netherlands; 25 Department of Medical Oncology, Amsterdam University Medical Centre, Free University Amsterdam, Amsterdam, the Netherlands; 26 Department of Pulmonology, Elisabeth-TweeSteden Hospital, Tilburg, the Netherlands; 27 Medical Advisor, Netherlands Health Care institute, Diemen, the Netherlands; 28 Department of Medical and Clinical Psychology, Center for Research on Psychological and Somatic Diseases (CoRPS), Tilburg University, Tilburg, the Netherlands; 29 Department of Psychosocial Research and Epidemiology, The Netherlands Cancer Institute, Amsterdam, the Netherlands; VIVES University of Applied Sciences - Campus Roeselare: Hogeschool VIVES - Campus Roeselare, BELGIUM

## Abstract

**Background:**

End-of-life care affects both patients with advanced cancer and their relatives but is often assessed from only one perspective, namely that of bereaved relatives. This study aimed to gain insight into the quality of care as experienced by patients with advanced cancer and their relatives.

**Methods:**

A total of 367 patients with stage IV cancer, 242 relatives and 163 bereaved relatives were included from a large prospective, longitudinal study (eQuiPe), which ran from November 2017 until March 2020. Patients and their relatives completed a questionnaire during the last 3 months of the patient’s life. Bereaved relatives completed a questionnaire within six months after the patient’s death.

**Results:**

At the end of life, patients reported a mean satisfaction with care score of 72/100 (SD 21), and relatives a mean score of 59/100 (SD 28) for the care they received themselves. Continuity with care, the extent to which the care received from different healthcare professionals was coordinated, was associated with higher satisfaction with care in patients (β 2.1, 95% CI 1.6–2.6). Bereaved relatives reported that most patients died peacefully (87%) and at home (73%). Most bereaved relatives (66%) were contacted by a healthcare professional after the patient’s death, but over half were not informed about grief (52%) or the available options for bereavement support (58%), with about 20% reporting they would have appreciated this.

**Conclusions:**

Quality of end-of-life care was generally perceived as good. This study highlights the importance of good continuity of care as it is associated with higher satisfaction with care in patients. Also, one-fifth of the bereaved relatives reported that they had not been informed about bereavement care despite desiring it, which emphasizes the need for better care for relatives before and after the patient’s death.

## Introduction

Early integration of palliative care in oncology improves the quality of end-of-life care, as it has been associated with better quality of life and a decreased likelihood of potentially inappropriate care [1–3]. End-of-life care is an important component of palliative care, aimed at ensuring patients’ comfort in the last phase of life [4]. Receiving potentially inappropriate care is not only associated with worse health outcomes in patients, such as a lower quality of life and higher burden of depressive symptoms, but also with higher hospital costs and increased likelihood of experiencing complicated grief and higher levels of regret in bereaved relatives [5–7]. Moreover, clinicians who provide appropriate end-of-life care and received expressions of gratitude experienced an enhanced perception of self-worth and professional value, which can serve as a motivational factor [8]. This shows that good end-of-life care is important for everyone who is involved in the disease trajectory, including patients, relatives, and clinicians. However, some challenges exist. For example, both patients and relatives still experience a high symptom burden, including physical pain, depression, intense emotions, loss of dignity and/or control and a feeling of hopelessness [9]. Moreover, they also have to deal with practical issues, such as financial or legal problems, causing additional distress [9,10].

The quality of end-of-life care can be assessed using quality indicators, which measure the level of potentially inappropriate end-of-life care and includes factors like the continuation of ongoing treatment very near to death, a high number of hospital admissions, emergency department admissions or intensive care unit days, and dying in an acute-care setting [11]. Although these quality indicators provide useful information, they do not reflect how patients and relatives experience the care they have received during the last part of the disease trajectory and, for relatives, also after death. One way to assess the quality of care from the patient or relative perspective is by measuring satisfaction with care. Multiple studies have shown that higher satisfaction with care is associated with better quality of life outcomes in both patients and their relatives [12–15]. Moreover, not only does satisfaction with care influence the quality of life in both patients and relatives separately, the quality of life of patients also affects that of their relatives and vice versa [16]. However, relatively little is known about which care-related factors contribute to higher satisfaction, particularly in the context of end-of-life care for patients with advanced cancer. Some studies suggest that receiving palliative care is associated with greater satisfaction in patients, while less aggressive care, such as fewer intensive care unit admissions and hospitalizations, is linked to higher satisfaction among relatives [17,18]. To gain a more comprehensive understanding of experienced quality of end-of-life care, more elaborate methods have been developed, including validated questionnaires such as the Views of Informal Carers’ Evaluation of Services (VOICES) and the Consumer Quality-index Palliative Care (CQ-index PC) [19,20].

Most studies using these questionnaires have reported only the perspective of bereaved relatives, included only small or specific populations, and are often retrospective in nature. To gain more insight into the experienced quality of care, including satisfaction with care, from the perspective of both patients and their relatives this study used data from a prospective, longitudinal, observational multicenter study (eQuiPe). Additionally, this study aimed to assess which factors were associated with higher satisfaction with end-of-life care in both patients and relatives.

## Materials and methods

### Study design

Data was used from a prospective, longitudinal, multicenter, observational study on experienced quality of care and quality of life in patients with advanced cancer and their relatives (eQuiPe). EQuiPe data were anonymized, linked to clinical data, and made available for research purposes as of February 1st, 2019; further details on the study protocol are published elsewhere [21]. In short, patients and relatives provided written consent, after which they were asked to complete a baseline questionnaire followed by 3-monthly follow-up questionnaires until the patients’ death. For this paper, the last questionnaire (completed less than three months before death) was used. Three to six months after the patients’ death, relatives were asked to complete an additional questionnaire on bereavement and their perspective on the quality of end-of-life care. The questionnaires could be completed on paper or online via the Patient Reported Outcomes Following Initial Treatment and Long-term Evaluation of Survivorship (PROFILES) registry [22]. Clinical data was retrieved by linking the questionnaire data to the Netherlands Cancer Registry (NCR). The Medical Research Ethics Committee of the Antoni van Leeuwenhoek Hospital (METC17.1491) exempted the eQuiPe study from medical ethical review, which was in accordance with the Dutch Medical Research Involving Human Subjects Act. The eQuiPe study is registered in the Netherlands Trial Register (NTR6584).

### Study population

Patients were invited by their treating physician in one of the forty participating hospitals or self-enrolled via an advertisement on a Dutch platform for patients and relatives who are confronted with cancer (www.kanker.nl) between November 2017 and March 2020. All patients aged ≥18 years, diagnosed with stage IV cancer and able to understand and complete a Dutch questionnaire were eligible for inclusion. The eQuiPe study aimed to assess the quality of life and quality of care in the last year of life of patients with advanced cancer. Therefore, additional criteria were applied to patients with breast and prostate cancer to exclude those with a relatively good prognosis. Patients with breast cancer were eligible if their cancer was metastasized in multiple organ systems and patients with prostate cancer were eligible if their cancer was castrate resistant. Relatives were invited by the patients and were eligible for inclusion when aged ≥18 years and able to understand and complete a Dutch questionnaire. Both patients and relatives were excluded from participation if they suffered from dementia or if they had a history of severe psychiatric illness. For this analysis, three distinct population groups were selected. First, patients with available quality of care data in the last three months of life were included to assess their satisfaction with care. Second, relatives with available data on the care they themselves received during the patient’s final three months were included to evaluate their own satisfaction with care. Third, bereaved relatives who completed a questionnaire within six months after the patient’s death were included to retrospectively report on the quality of end-of-life care provided to the patient, as well as their own experiences with care during and after the dying phase. Patients and relatives could participate independently in the eQuiPe study, meaning that not every patient had a participating relative and vice versa. Additionally, due to the prospective nature of the study, not all participants completed questionnaires within the final three months of life, and not all relatives completed the post-death questionnaire. As a result, there is partial overlap between the group of relatives and bereaved relatives, but these groups are not identical. The eQuiPe study allowed multiple (bereaved) relatives per patient to participate. However, to ensure that each end-of-life care situation was represented equally and to avoid overrepresentation of individual cases, only one bereaved relative per patient was included in this analysis. Including multiple bereaved relatives for a single patient could have introduced imbalance, as some care experiences would be reflected more than once. In four cases, data from two bereaved relatives were available: three partner-child pairs and one child-child pair. For the partner-child pairs, we selected the relative who was closest to the patient (the partner). For the child-child pair, the child who completed the questionnaire closest to the patients’ death was included to minimize potential recall bias.

### Measurements

#### Healthcare utilization.

Healthcare use was measured using a self-composed questionnaire. Patients and relatives were asked how often and which healthcare professional they had contacted in the previous month. Healthcare professionals included in the response options were general practitioner, medical specialist, nurse specialist, home care nurse, consultant of specialist palliative care team, psychologist, sexologist, social worker, spiritual caregiver, physiotherapist, occupational therapist, dietician and other. The number of contacts and the number of different healthcare professionals were summed up to a total number of healthcare contacts and a total number of different healthcare professionals contacted in the previous month.

#### Satisfaction with care.

For both patients and relatives, satisfaction with care was measured using the validated in-patient Cancer Care questionnaire (EORTC IN-PATSAT32) [23]. This included one item on general satisfaction with care, and five subscales evaluating the healthcare professionals providing the care they received (interpersonal skills, availability, empathic skills, technical skills, and information provision). Satisfaction with care and all five subscales were answered on a 5-point Likert scale and were linearly transformed to a continuous score ranging from 0 to 100, with higher scores indicating higher satisfaction with care.

#### Continuity of care and contradictory information.

For patients, the experienced continuity of care (“Does the care you received from different care providers match each other?”) and receiving contradictory information (“Did the information you were given from different care providers contradict each other?”) were measured using single items from the Consumer Quality Index Palliative Care [24]. Both continuity with care and contradictory information, were answered on a 4-point Likert scale and linearly transformed to a continuous score ranging from 0–100, with higher scores indicating better continuity with care and less contradictory information received.

#### Caregiver burden.

Caregiver burden experienced by relatives was measured using the validated short-form version of the Zarit Caregiver Burden Interviews (ZBI-12) [25,26]. The ZBI-12 consists of 12 items, which were answered on a 5-point Likert scale ranging from 0“never” to 4“nearly always”. The total score ranged from 0 to 48, with higher scores indicating a higher caregiver burden.

#### Experienced end-of-life care.

Patient’s experienced level of peace was measured using the peace subscale from the Dutch Functional Assessment of Chronic Illness Therapy – Spiritual Well-Being 12 item Scale (FACIT-Sp-12) [27]. This instrument assesses spiritual well-being across three dimensions: peace, meaning, and faith. The peace subscale specifically reflects a person’s sense of inner peace, acceptance, and harmony with the situation. Originally this subscale included four items but a validation study in patients with advanced cancer showed acceptable measurement properties for only three of the four items [28]. Therefore, this study included the three validated items, which were answered on a 5-point Likert scale and summed up into a score ranging from 0–12 with higher scores indicating more peace.

The perspective of bereaved relatives on the care the patient received was measured within six months after the patient died and existed of validated questions combined with self-composed items. It comprised three time periods: patients ‘care in the last three months of life, in the last seven days of life and circumstances at the time of the patients’ death. Care in the last three months of the patients’ life was measured using the Views of Informal Carers’ Evaluation of Services Short Form (VOICES-SF) [20]. Care in the last seven days of the patients’ life were assessed using five self-composed items. Three of these items were about the patient (“Did the patient know death was near?”, “Did the patient have peace with his/hers nearing death?” and ‘Was the patient afraid to die?”) and two were related to the healthcare professional’s communication towards the patients (“Did the healthcare professional inform the patient about their nearing death in a tactful manner?” and “Was the patient treated with respect and dignity?”). Circumstances around the patient’s death was measured using four additional self-composed items. Two items were related to the patient’s place of death (“Where did the patient die?” and “Was the patient able to choose his/her own place of death?”) and two items were related to the circumstances of the patient at the moment of death (“Did the patient die peacefully?” and “Did it seem like the pain of the patient was under control during the moment of death?”).

#### Bereavement care.

Within six months after the patient died, bereaved relatives were also asked about the care they had received for themselves during and after the patient’s death using self-composed questions. Five items were included on the care they had received just before or during the patients’ death; “Were you contacted in time to be present when the patient died?”, “Were you given enough space to say goodbye properly?”, “Were you and other relatives given enough support at the time of death?”, “Have you been informed about grief and loss and which feelings it may include?” and “Have you been informed about available support options for grief and loss, such as peer support or professional counseling?””). Additionally, two items were included about the care they had received after the patients had died; “Did care professionals treat you tactfully after the patient had died?” and “Since the patient’s death, have you discussed the illness trajectory or the death with a healthcare professional?”). The response options for most of the items included “yes”, “no”, or “I do not know”. Except for the items on being informed about grief and loss, the available support options for grief and loss and if they had spoken to a healthcare professional after the patient’s death, those included the following response options: “Yes”, “No, but I would have liked it”, “No, but I was not interested anyway” and “I do not know”.

#### Socio-demographic and clinical characteristics.

Date of birth, gender and level of education were included in the baseline questionnaire for both patients and relatives. Age was calculated as the number of years between the date of birth and the date of completion of the questionnaire. The level of education was categorized as low, medium, and high according to the guidelines of the International Standard Classification of Education. Relatives also completed an item in the baseline questionnaire about the type of relationship they have with the patient (partner, child, family, friend or other). Comorbidity was measured in the baseline questionnaire of the patients, using the Self-administered Comorbidity Questionnaire (SCQ) [19,29]. Patients’ date of death, date of primary diagnosis, primary tumor type and whether their cancer had metastasized at primary diagnosis were obtained from the NCR.

### Statistical analysis

Descriptive analyses were performed to summarize the sociodemographic and clinical characteristics of both patients and relatives. Boxplots were used to summarize and visualize the satisfaction of care in both patients and relatives. Additionally, linear regression analyses were performed to explore which factors were associated with higher satisfaction with care in both patients and relatives. Experienced end‑of‑life care from the perspective of bereaved relatives was described, and differences were examined using Fisher’s exact tests in univariate stratified analyses by place of care (during the last seven days of life), place of death, age (<65, 65–75, ≥ 75) and gender. All statistical analyses were performed using STATA version 17.0. Statistical significance was set at p < 0.05 for the regression analyses and at p < 0.01 for the univariate analyses to correct for multiple testing.

## Results

**Fig 1:**
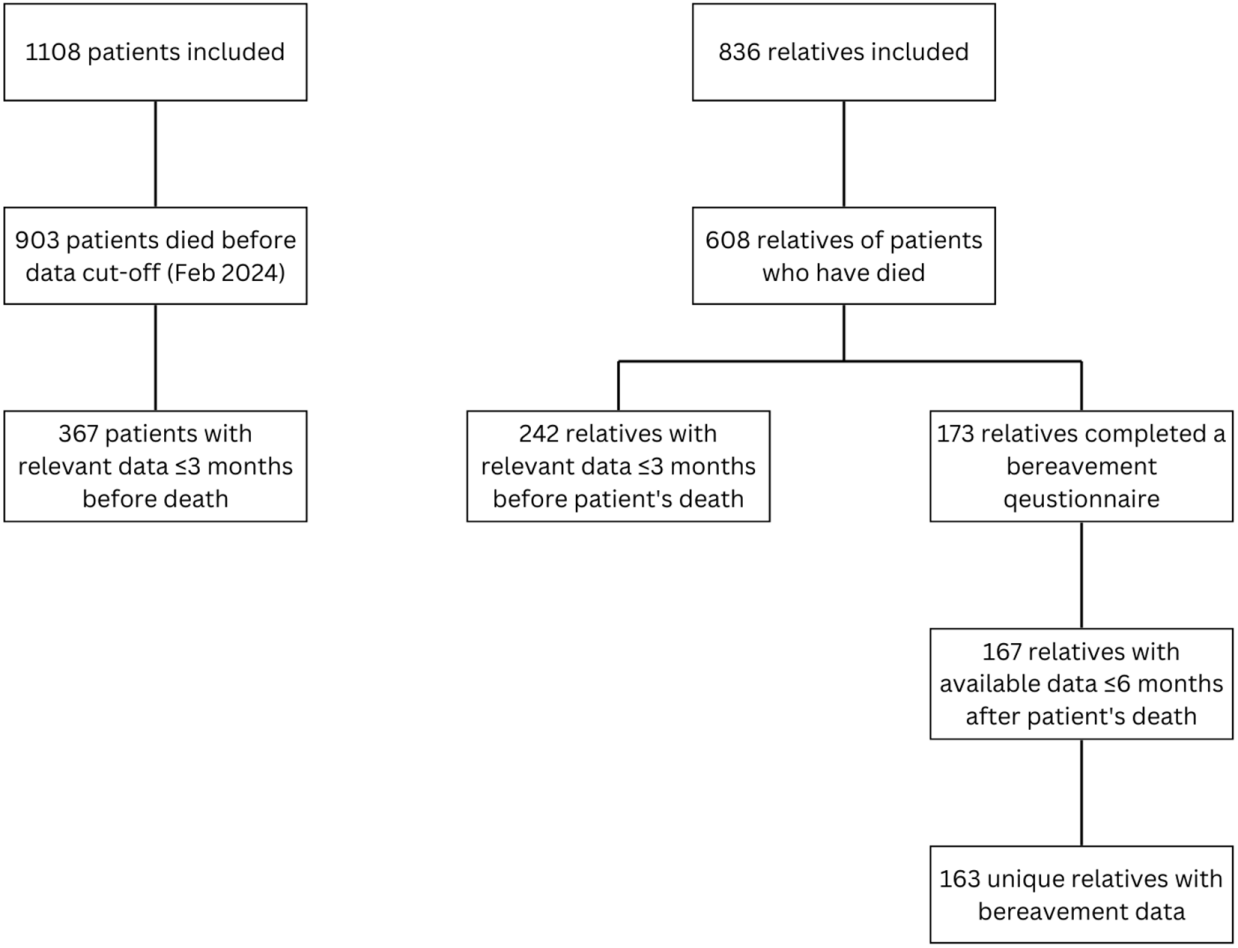
Flowchart of the inclusion and exclusion of patients, relatives and bereaved relatives.

In total, 367 patients and 242 relatives had available satisfaction with care data during the last three months of the patients’ life and were included in this analysis (Fig 1). In addition, 163 bereaved relatives completed a questionnaire within six months after the patient’s death.

### Sociodemographic and clinical characteristics

Patients had a mean age of 66 (SD 9) years, and 50% were female (Table 1). The majority reported having at least one comorbidity (65%), and the most common primary cancer types were lung (31%), colorectal (18%) and breast (14%) cancer. The mean age of relatives with available data about the care they received during the last three months of the patients’ life was 62 (SD 12) years, 60% were female, and most were the partner of the patient (78%). Similarly, bereaved relatives had a mean age of 61 (SD 12) years, 57% were female, and the majority of them were the partner of the patient (80%) (Table 1)

**Table 1:** Socio-demographic and clinical characteristics of patients with advanced. cancer and their relatives.

	Patients (n = 367)	Relatives (n = 242)	Bereaved relatives (n = 163)
Age (mean (SD))	66 (SD 9)	62 (SD 12)	61 (SD 12)
	N (%)	N (%)	N (%)
**Gender**			
*Female*	182 (50)	146 (60)	94 (58)
*Male*	182 (50)	93 (38)	69 (42)
*Missing*	3 (1)	3 (1)	–
**Education level**			
*Low*	109 (30)	63 (26)	30 (18)
*Medium*	147 (40)	96 (40)	72 (44)
*High*	106 (29)	78 (32)	61 (37)
*Missing*	5 (1)	5 (2)	–
**Relationship with patient**			
*Partner*	–	188 (78)	130 (80)
*Child*	–	31 (13)	21 (13)
*Other*	–	18 (7)	10 (6)
*Missing*		5 (2)	2 (1)
**Comorbidities (% yes)**			
*No*	118 (32)	–	–
*One*	126 (34)	–	–
*More than one*	115 (31)	–	–
*Missing*	8 (2)		
**Cancer type** ^**a**^			
*Lung*	114 (31)	–	–
*Colorectal*	66 (18)	–	–
*Breast*	50 (14)	–	–
*Prostate*	27 (7)	–	–
*Other*	108 (29)	–	–
*Missing*	2 (1)		
**Time since diagnosis** *(years)*	3.6 (SD 4.8)	–	–
**Metastasis at primary diagnosis** (%yes)	186 (51)	–	–

^*a*^Other cancer types included upper gastrointestinal cancer (n = 16), pancreatic cancer (n = 8), urological cancer (n = 8), gynecological cancer (n = 6) or other types of cancer (n = 70).

### End-of-life care for patients with advanced cancer

#### From the perspective of patients.

**Fig 2:**
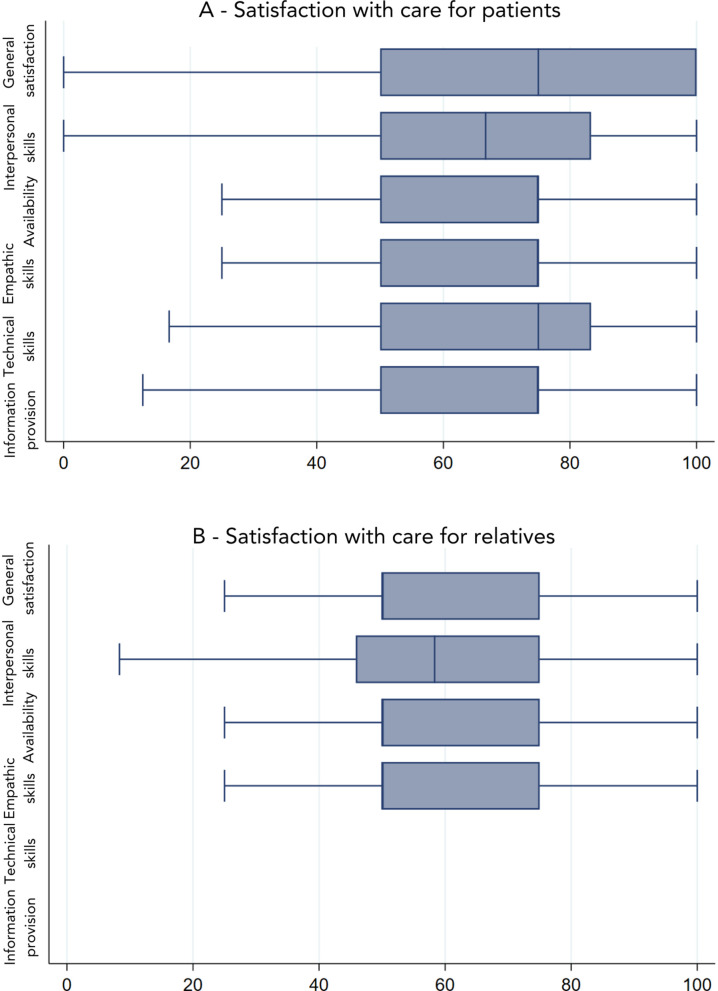
Mean satisfaction with care in the last three months of the patient’s life reported by patients with advanced cancer and their relatives.

At the end of life, most patients were relatively at peace with their situation (mean score of 8 out of 12 (SD 3)). Patients reported having contacted a median of three (IQR 2–4) different healthcare professionals and a median of six (IQR 3–11) healthcare contacts in the previous month. The most reported healthcare professional they had contacted was their medical specialist (81%), followed by their general practitioner (72%), nurse specialist (59%) and home care (25%). Seven percent of patients reported to have been in contact with a consultant from a palliative care team. Patients reported a mean score of 72 (SD 21) for general satisfaction with the care they had received in the previous month (Fig 2). Multivariable regression analysis showed that patients’ general satisfaction with care increased with greater experienced continuity of care (β 2.1, 95% CI 1.6; 2.6) and decreased when receiving contradictory information from healthcare professionals (β 1.0, 95% CI 0.4; 1.6) (S1 Table).

#### From the perspective of bereaved relatives.

During the last seven days of the patients’ life, bereaved relatives reported that their patient was cared for at home (74%), in hospital (14%) or in a hospice (10%). According to bereaved relatives, 87% of patients were aware that their death was imminent; this percentage was lower in patients who stayed in hospital (65%) compared to those who stayed at home (90%) or in a hospice (94%, p = 0.003) (Table 2). Relatives reported that 69% of the patients were at peace with his or her approaching death and that 19% was afraid to die, which did not significantly differ between places of care. Bereaved relatives reported that 73% of the patients died at home, 17% in hospital and 10% in hospice care. In the last few days of life, four patients were moved from their homes to the hospital where three of them died. According to the relatives, pain seemed to be under control in 79% of the patients and 87% of the patients died peacefully (Table 3). Stratified analyses by gender and age showed no significant differences in experienced end‑of‑life care before, during or after the patient’s death (S3-S5 Tables).

**Table 2:** Experienced end-of-life care for patients with advanced cancer in their last week of life from the perspective of bereaved relatives (n = 163) stratified for place of care.

	Total (N = 163) N (%)	Home (n = 120) N (%)	Hospital (n = 23) N (%)	Hospice (n = 16) N (%)	p-value^a^
**Did the patient know death was near?** (% yes)	141 (87)	108 (90)	15 (65)	15 (94)	**0.003**
**Did the care professional inform the patient about their nearing death in a tactful manner?**					0.447
*Very tactful*	108 (66)	82 (68)	12 (52)	12 (75)	
*A little tactful*	27 (17)	20 (17)	4 (17)	2 (13)	
*Not at all tactful*	6 (4)	4 (3)	1 (4)	1 (6)	
*Not informed*	6 (4)	4 (3)	1 (4)	1 (6)	
*Unexpected*	10 (6)	6 (5)	4 (17)	–	
*Unknown*	6 (4)	4 (3)	1 (4)	–	
**Was the patient treated with respect and dignity?**					**0.005**
*All the time*	137 (84)	106 (88)	15 (65)	13 (81)	
*Most of the time*	20 (12)	13 (11)	4 (17)	3 (19)	
*Sometimes*	3 (2)	–	3 (13)	–	
*Never*	–	–	–	–	
*Unknown or missing*	3 (2)	1 (1)	1 (4)	–	
**Did the patients have peace with their nearing death?** (% yes)	112 (69)	86 (72)	17 (74)	8 (50)	0.058
**Was the patient afraid to die?** (% yes)	31 (19)	20 (17)	6 (26)	4 (25)	0.173

Percentages may not add up to 100% due to rounding and four patients had either missing data or specified their place of care as “other” and were not included in the stratified analysis.

^a^P-values of <0.01 were deemed statistically significant

**Table 3:** Relatives’ experiences of the quality of care during and after the death of patients with advanced cancer (n = 163), stratified for place of death.

	Total (n = 163)	Home (n = 119)	Hospital (n = 27)	Hospice (n = 16)	
	N (%)	N (%)	N (%)	N (%)	p-value^a^
Quality of end-of-life care and dying					
**Was the patient able to choose their own location of death?**					**<0.001**
*Yes*	133 (82)	112 (93)	8 (30)	13 (81)	
*No*	9 (6)	1 (1)	6 (22)	2 (13)	
*I do not know*	8 (5)	2 (2)	4 (15)	1 (6)	
*Patient died suddenly*	13 (8)	4 (3)	9 (33)	–	
**Did it seem like the pain of the patient was under control?** (%yes)	129 (79)	97 (80)	18 (67)	13 (81)	0.235
**Did the patient die peacefully?** (%yes)	141 (87)	105 (87)	22 (81)	14 (88)	0.389
End-of-life care and bereavement care for the relatives					
**Where you contacted on time so you could be there when the patient died?**					0.029
*Yes, I was contacted on time or was already with the patient*	147 (90)	111 (92)	22 (79)	13 (81)	
**Did you and other relatives received sufficient support at the moment of death?**					0.988
*Very much*	120 (74)	87 (72)	18 (64)	13 (81)	
*A little bit*	28 (17)	20 (17)	6 (21)	2 (13)	
*Not at all*	10 (6)	7 (6)	2 (7)	1 (6)	
*Unknown or missing*	5 (3)	5 (4)	–	–	
**Did you receive enough space to properly say goodbye?** (%yes)	152 (93)	112 (93)	23 (82)	15 (94)	0.118
**Did care professionals treat you tactfully after the patient died?** (%yes)	150 (92)	112 (93)	22 (79)	14 (88)	0.130

Percentages may not add up to 100% due to rounding and four patients had either missing data or specified their place of death as “other” and were not included in the stratified analysis.

^a^P-values of <0.01 were deemed statistically significant.

### Quality of care for relatives themselves

#### During the last three months of the patient’s life.

Fifty-two percent of the relatives reported that they had not received any care for themselves during the last three months of the patient’s life. Relatives who did receive care for themselves reported having contacted a healthcare professional with a median of 3 (IQR 1–7) times and a median of 2 (IQR 1–3) different healthcare professionals. The healthcare professional they most often had contact with was the medical specialist (33%) with a median number of two (IQR 1–3) contacts in the previous month. Relatives who had received care in the last three months of the patient’s life reported a mean general satisfaction with care of 59 (SD 28) (Fig 2). Multivariable regression analysis showed that higher caregiver burden (β −6.8, 95% CI −10.6; −3.1) was significantly associated with lower general satisfaction with care in relatives (S1 Table).

#### During and after the patient’s death.

**Fig 3:**
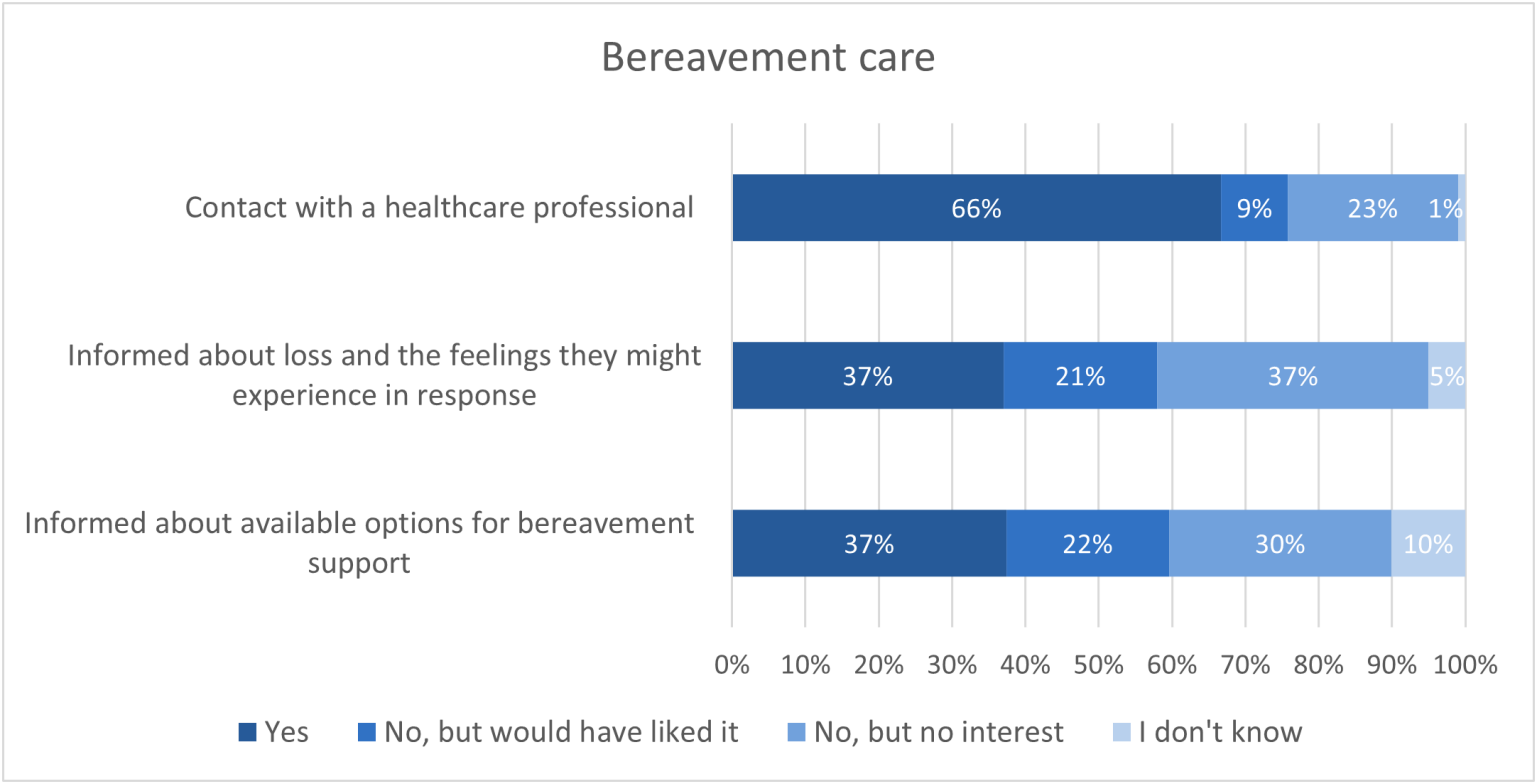
Bereavement care in relatives (n = 163) of patients with advanced cancer.

Ninety percent of the bereaved relatives reported that they had been contacted in time or were already with the patient when the patient was dying and 93% reported that they received enough space to properly say goodbye (Table 3). Thirty-seven percent had received information about loss and grief and 37% about available options for bereavement support. Approximately one-fifth of bereaved relatives would have liked to be offered information about loss and grief (21%) or bereavement support (22%) but had not received any. Sixty-six percent of bereaved relatives reported to have spoken about the illness trajectory or death with a healthcare professional at least once since the patient had died (Fig 3).

## Discussion

This study shows that the quality of end-of-life care was perceived as good by the great majority of both patients with advanced cancer and their relatives. Multivariable regression analyses showed that greater continuity of care and less contradictory information were associated with higher patient satisfaction, while a higher caregiver burden was associated with lower satisfaction in relatives. Most relatives reported that patients died peacefully and at home. However, one-fifth of bereaved relatives reported not receiving information about grief or available bereavement support, despite desiring it.

Patients in our study were relatively satisfied with the care they received during the last three months of life, which was associated with continuity of care and information provision. While high satisfaction (67%−89%) with end-of-life cancer care reported by bereaved relatives has also been observed within multiple European, American and Canadian studies [30–37], patient perspectives are far less frequently examined. One study reporting on the perspectives of Dutch patients with advanced cancer, reported similar findings regarding general satisfaction and its subdomains: 72 (SD 21) for general satisfaction, 67 (SD 24) for interpersonal skills, 62 (SD 22) for availability, 68 (SD 22) for technical skills and 66 (SD 24) for information provision, respectively [12]. The importance of information provision and continuity of care in relation to higher satisfaction has also been supported by recent studies. For example, a Chinese study in 213 patients with advanced breast cancer found that unmet health information needs were associated with lower satisfaction during the first year after diagnosis [38]. In addition, a Canadian study of 2790 patients receiving chemotherapy and/or radiotherapy identified the four most relevant predictors of satisfaction; being informed about follow-up, understanding the next step in the disease trajectory, knowing whom to contact with questions, and clinicians’ awareness of all test results [39]. These findings underscore the critical role of adequate information provision and continuity of care in enhancing patients’ satisfaction with care, also at the end of life.

While patients in our study generally reported high satisfaction with end‑of‑life care, relatives’ own satisfaction with the care they personally received was considerably lower (mean 59/100). Few studies have examined satisfaction in relatives of patients with advanced cancer, and little is known about the healthcare use of relatives during this specific time period. A Swiss study found that recently bereaved relatives of cancer patients were reasonably satisfied with the support provided by nurses during the end-of-life of their loved ones (median score of 50, with a total score range 14–70) [40]. In addition, our study showed that a higher caregiver burden was significantly associated with lower satisfaction with care. This finding is consistent with existing research showing that relatives often prioritize caregiving responsibilities over their own health [41–44], and with a Dutch cross‑sectional study demonstrating that higher caregiver burden was associated with lower self‑care and resilience [45]. Literature further indicates that supporting family members can reduce caregiver burden and improve their quality of life [46–49]. These findings emphasize the importance of healthcare professionals actively assessing and addressing relatives’ needs, as they are unlikely to seek help themselves.

Overall, the quality of end‑of‑life care was perceived as good by both patients and bereaved relatives, but some differences between places of death (home, hospital, hospice) were found on individual items reported by bereaved relatives. Bereaved relatives generally reporting more positive experiences when patients died at home, which aligns with previous studies showing that home death is associated with better symptom control, improved death preparedness, higher overall quality of dying, and a lower risk of prolonged grief among relatives [50–53]. Hospital deaths are often perceived as less favorable, possibly due to the impersonal and less peaceful environment. For example, the lack of single rooms may compromise privacy, and relatives often face difficulties gaining the attention of healthcare professionals and understanding clinical information due the use of medical jargon in hospitals [54]. Our study also showed that 33% (n = 9) of bereaved relatives of whom the patient had died in hospital indicated that death was sudden, and 17% (n = 4) of these relatives also reported that the healthcare professionals were unaware of the patient’s impending death. This suggests that patients may have deteriorated quickly resulting in an earlier-than-expected death and hospitalization at the end of life. Hospitalization at the end of life is often associated with delayed access to specialized palliative care and poor integration of advance care planning [55–59], both of which negatively affect patients’ death preparedness and relatives’ perspectives on the quality of end-of-life care [60,61]. These findings highlight the need for timely discussions about illness severity and death, as well as early integration of palliative care and advance care planning to improve quality of life and reduce avoidable hospital admissions. As not all hospital deaths are preventable, strengthening hospital‑based care by ensuring privacy and clear, attentive communication remains essential. Importantly, experiences of hospital death are not universally negative, underscoring the importance of tailoring improvements to individual needs and circumstances across all care settings [62].

Although care professionals agree on the importance of offering aftercare to bereaved relatives [66], our study shows that only one-third of bereaved relatives were informed about grief and bereavement support. Moreover, one-fifth reported not having received such information, despite desiring it. Additionally, two-thirds of relatives had discussed the illness trajectory and death with a healthcare professional since the patient’s death. The number of studies reporting the proportion of relatives receiving any form of bereavement care is limited, but available data suggests that this proportion is slightly higher, ranging between 50% and 77% [63–65]. Various forms of bereavement care exist, and their implementation may differ across healthcare settings. For example, a Dutch study showed that general practitioners contacted bereaved relatives at least once in 81% of cases following death whereas a recent review reported that follow-up calls were less routinely integrated in hospitals [66,67]. Barriers such as limited time, training, and organizational support can hinder the provision of aftercare [66,68]. Moreover, bereavement care can be provided by a range of professionals, each playing distinct and complementary roles and therefore should be approached as a shared responsibility, with clear communication within interdisciplinary teams to ensure no one is overlooked. [40,69]. In addition, adequate training, time, and structural support across care settings are essential to provide meaningful, coordinated aftercare that supports relatives’ long‑term wellbeing.

This unique prospective, longitudinal, observational multicenter study enabled us to examine the perceived quality of care and satisfaction with care both before and after the patient’s death, from the perspectives of patients and relatives. Nevertheless, several limitations should be acknowledged. Selection bias may have influenced our findings, as participants with better health and higher educational attainment are more likely to enroll in survey studies [70]. In addition, our sample was relatively homogeneous, with over 90% of participants of Dutch origin and most reporting Catholic/Protestant or no religious affiliation. Individuals from minority and migrant groups are often less integrated into healthcare and less likely to join research, which may partly explain high satisfaction scores. Moreover, their underrepresentation limits generalizability, as cultural and religious contexts shape care experiences [71–73]. For example, patients’ cultural and spiritual backgrounds may influence how they interpret illness, what their communication preferences are, and how they evaluate the appropriateness of care, which may lead to systematically different experiences among underrepresented groups. These dynamics underscore the importance of capturing diverse perspectives to better understand variation in satisfaction and continuity of care. Future studies should adopt active inclusion strategies to better engage these populations and ensure more representative insights. Recall bias may have occurred, as bereaved relatives reported experiences three to six months after death. Despite our attempt to limit this by restricting inclusion to within six months, some degree of under- or overestimation remains possible. Follow-up partially took place during the COVID-19 pandemic. While the pandemic did not significantly affect oncological hospital care for patients with advanced cancer in the Netherlands [74,75], it may have negatively impacted their relatives. Reports from Dutch primary care indicated postponed help-seeking behavior, reduced accessibility, and decreased continuity of care [76–78], and certain death-related rituals could not be performed due to government restrictions [79,80]. These barriers may have limited the support relatives received, lowered satisfaction with care, and shaped their experiences of dying and bereavement. Moreover, the eQuiPe study focused on the final phase of life rather than long‑term bereavement outcomes, conclusions about the effectiveness of bereavement support cannot be drawn, underscoring the need for future research. Finally, the variation in assessment tools complicates cross‑study and cross‑national comparisons. While VOICES is a well-established and validated retrospective tool for capturing bereaved relatives’ perspectives, there remains a need to develop a universal gold standard applicable across various settings to ensure comparability.

## Conclusion

This study shows that most patients with advanced cancer and their relatives generally perceived the quality of end-of-life care to be good. At the same time, it emphasizes the importance of continuity of care, as higher perceived continuity was associated with greater satisfaction with care in patients. Furthermore, a substantial proportion of relatives reported not being informed about grief and the available bereavement support options despite desiring it. Given that multiple healthcare professionals are often involved, it is essential to ensure well-coordinated care across settings to meet the needs of relatives, both during and after the patient’s death.

## Supporting information

S1 Table Multivariable linear regression analyses assessing which factors contribute to the general satisfaction with care in the last 3 months of the patients’ life experienced by patients (n = 291) and relatives n = 100). (DOCX)

S2 Table Experienced end-of-life care for patients with advanced cancer in their last week of life from the perspective of bereaved relatives (n = 163) stratified by gender. (DOCX)

S3 Table Relatives’ experiences (n = 163) of the quality of care during and after the patient’s death, stratified by gender. (DOCX)

S4 Table Experienced end-of-life care for patients with advanced cancer in their last week of life from the perspective of bereaved relatives (n = 163) stratified by age. (DOCX)

S5 Table Relatives’ experiences (n = 163) of the quality of care during and after the patient’s death, stratified by age. (DOCX)
