# Supplementary material for: Patients’ and relatives’ perspectives on the quality of end-of-Life care in advanced cancer: From the final months to bereavement
Source: PLoS One. 2026 Feb 9;21(2):e0342068. doi: 10.1371/journal.pone.0342068 (PMC12885308; doi:10.1371/journal.pone.0342068)
Supplement: S1 Table — (DOCX) [file pone.0342068.s001.docx]

**S1 Table: Multivariable linear regression analyses assessing which factors contribute to the general satisfaction with care in the last 3 months of the patients’ life experienced by patients (n=291) and relatives n=88).**

|  | Patients  (n=291) | | | Relatives  (n=88) | | |
| --- | --- | --- | --- | --- | --- | --- |
|  | Beta | 95% CI | p-value | Beta | 95% CI | p-value |
| **Age** | -0.03 | -0.3 ; 0.2 | 0.820 | -0.37 | -1.0 ; 0.3 | 0.260 |
| **Gender** |  |  |  |  |  |  |
| *Male* | Ref | Ref |  | Ref | Ref |  |
| *Female* | 1.72 | -3.4 ; 6.8 | 0.507 | 5.45 | -6.3 ; 17.2 | 0.357 |
| **Education** |  |  |  |  |  |  |
| *Low* | Ref | Ref |  | Ref | Ref |  |
| *Medium* | 0.04 | -5.4 ; 5.5 | 0.989 | 1.57 | -12.5; 15.6 | 0.825 |
| *High* | 0.59 | -5.3 ; 6.4 | 0.843 | 0.54 | -16.2; 17.2 | 0.949 |
| **Cancer type** |  |  |  |  |  |  |
| *Lung* | Ref | Ref |  |  |  |  |
| *Colorectal* | 2.50 | -4.3 ; 9.3 | 0.468 | - | - |  |
| *Breast* | 0.10 | -8.9 ; 9.1 | 0.983 | - | - |  |
| *Prostate* | -9.27 | -19.5 ; 1.0 | 0.077 | - | - |  |
| *Other* | -2.21 | -8.0 ; 3.5 | 0.448 | - | - |  |
| **Received cancer-specific treatment** |  |  |  |  |  |  |
| *No* | Ref | Ref |  | - | - |  |
| *Yes* | 3.29 | -2.9 ; 9.5 | 0.300 | - | - |  |
| **Time between diagnosis and death** *(years)* | 0.01 | -0.5 ; 0.6 | 0.980 | - | - |  |
| **Continuity of care** *(per 5)* | **2.07** | **1.6 ; 2.6** | **<0.001** | **-** | **-** |  |
| **Contradictory information** *(per 5)* | **0.98** | **0.4 ; 1.6** | **0.001** | - | - |  |
| **Caregiver burden** *(per 5)* | **-** | **-** | **-** | **-6.80** | **-10.5 ; -3.1** | **0.001** |
