# Supplementary material for: Patients’ and relatives’ perspectives on the quality of end-of-Life care in advanced cancer: From the final months to bereavement
Source: PLoS One. 2026 Feb 9;21(2):e0342068. doi: 10.1371/journal.pone.0342068 (PMC12885308; doi:10.1371/journal.pone.0342068)
Supplement: S2 Table — (DOCX) [file pone.0342068.s002.docx]

**S2 Table. Experienced end-of-life care for patients with advanced cancer in their last week of life from the perspective of bereaved relatives (n=163) stratified by gender.**

|  | Male  patients  (N=69)  N (%) | Female  patients  (n=76)  N (%) | p-value^a^ |
| --- | --- | --- | --- |
| **Did the patient know death was near?** (% yes) | 54 (78) | 72 (95) | 0.011 |
| **Did the care professional inform the patient about their nearing death in a tactful manner?** |  |  | 0.037 |
| *Very tactful* | 41 (59) | 58 (76) |  |
| *A little tactful* | 11 (17) | 12 (16) |  |
| *Not at all tactful* | 3 (4) | 2 (3) |  |
| *Not informed* | 3 (4) | 1 (1) |  |
| *Unexpected* | 9 (13) | 1 (1) |  |
| *Unknown* | 1 (1) | 2 (3) |  |
| **Was the patient treated with respect and dignity?** |  |  | 0.847 |
| *All the time* | 57 (83) | 66 (87) |  |
| *Most of the time* | 8 (12) | 9 (12) |  |
| *Sometimes* | 2 (3) | 1 (1) |  |
| *Never* | - | - |  |
| *Unknown or missing* | 2 (3) | - |  |
| **Did the patients have peace with their nearing death?** (% yes) | 46 (67) | 56 (74) | 0.461 |
| **Was the patient afraid to die?** (% yes) | 17 (25) | 8 (11) | 0.043 |

^a^P-values of <0.01 were considered statistically significant.
