# Supplementary material for: Patients’ and relatives’ perspectives on the quality of end-of-Life care in advanced cancer: From the final months to bereavement
Source: PLoS One. 2026 Feb 9;21(2):e0342068. doi: 10.1371/journal.pone.0342068 (PMC12885308; doi:10.1371/journal.pone.0342068)
Supplement: S3 Table — (DOCX) [file pone.0342068.s003.docx]

**S3 Table. Relatives’ experiences (n=163) of the quality of care during and after the patient’s death, stratified by gender.**

|  | Male patients (n=69) | Female patients (n=76) |  |
| --- | --- | --- | --- |
|  | N (%) | N (%) | p-value^a^ |
| Quality of end-of-life care and dying |  |  |  |
| **Was the patient able to choose their own location of death?** |  |  | 0.087 |
| *Yes* | 54 (78) | 67 (88) |  |
| *No* | 2 (3) | 4 (5) |  |
| *I do not know* | 4 (6) | 3 (4) |  |
| *Patient died suddenly* | 9 (13) | 2 (3) |  |
| **Did it seem like the pain of the patient was under control?** (%yes) | 55 (80) | 61 (80) | 0.902 |
| **Did the patient die peacefully?** (%yes) | 61 (88) | 66 (87) | 0.926 |
| End-of-life care and bereavement care for the relatives |  |  |  |
| **Where you contacted on time so you could be there when the patient died?** |  |  | 0.811 |
| *Yes, I was contacted on time or was already with the patient* | 62 (90) | 70 (92) |  |
| **Did you and other relatives received sufficient support at the moment of death?** |  |  | 0.499 |
| *Very much* | 54 (78) | 57 (75) |  |
| *A little bit* | 9 (13) | 13 (17) |  |
| *Not at all* | 3 (4) | 5 (7) |  |
| *Unknown or missing* | 3 (4) | 1 (1) |  |
| **Did you receive enough space to properly say goodbye?** (%yes) | 66 (96) | 71 (93) | 1.000 |
| **Did care professionals treat you tactfully after the patient died?** (%yes) | 64 (93) | 71 (93) | 0.066 |

^a^P-values of <0.01 were considered statistically significant.
