# Supplementary material for: Patients’ and relatives’ perspectives on the quality of end-of-Life care in advanced cancer: From the final months to bereavement
Source: PLoS One. 2026 Feb 9;21(2):e0342068. doi: 10.1371/journal.pone.0342068 (PMC12885308; doi:10.1371/journal.pone.0342068)
Supplement: S4 Table — (DOCX) [file pone.0342068.s004.docx]

**S4 Table. Experienced end-of-life care for patients with advanced cancer in their last week of life from the perspective of bereaved relatives (n=163) stratified by age.**

|  | ≤64  years (n=59)  N (%) | 65-74 years  (n=58)  N (%) | ≥75  years  (n=28)  N (%) | p-value^a^ |
| --- | --- | --- | --- | --- |
| **Did the patient know death was near?** (% yes) | 55 (93) | 45 (78) | 26 (93) | 0.144 |
| **Did the care professional inform the patient about their nearing death in a tactful manner?** |  |  |  | 0.059 |
| *Very tactful* | 43 (73) | 35 (60) | 21 (75) |  |
| *A little tactful* | 11 (18) | 9 (16) | 3 (11) |  |
| *Not at all tactful* | 2 (3) | 1 (2) | 2 (7) |  |
| *Not informed* | 2 (3) | 1 (2) | 1 (4) |  |
| *Unexpected* | 1 (2) | 9 (16) | - |  |
| *Unknown or missing* | - | 3 (5) | 1 (4) |  |
| **Was the patient treated with respect and dignity?** |  |  |  | 0.366 |
| *All the time* | 51 (86) | 48 (83) | 24 (86) |  |
| *Most of the time* | 8 (14) | 5 (9) | 4 (14) |  |
| *Sometimes* | - | 3 (5) | - |  |
| *Never* | - | - | - |  |
| *Unknown or missing* | - | 2 (3) | - |  |
| **Did the patients have peace with their nearing death?** (% yes) | 40 (68) | 42 (72) | 20 (71) | 0.159 |
| **Was the patient afraid to die?** (% yes) | 18 (31) | 6 (10) | 1 (4) | 0.012 |

^a^P-values of <0.01 were considered statistically significant.
