# Supplementary material for: Patients’ and relatives’ perspectives on the quality of end-of-Life care in advanced cancer: From the final months to bereavement
Source: PLoS One. 2026 Feb 9;21(2):e0342068. doi: 10.1371/journal.pone.0342068 (PMC12885308; doi:10.1371/journal.pone.0342068)
Supplement: S5 Table — (DOCX) [file pone.0342068.s005.docx]

**S5 Table. Relatives’ experiences (n=163) of the quality of care during and after the patient’s death, stratified by age.**

|  | ≤64  years (n=59)  N (%) | 65-74 years  (n=58)  N (%) | ≥75  years  (n=28)  N (%) | p-value^a^ |
| --- | --- | --- | --- | --- |
| Quality of end-of-life care and dying |  |  |  |  |
| **Was the patient able to choose their own location of death?** |  |  |  | 0.034 |
| *Yes* | 49 (83) | 47 (81) | 25 (89) |  |
| *No* | 3 (5) | 3 (5) | - |  |
| *I do not know* | 4 (7) | - | 3 (11) |  |
| *Patient died suddenly* | 3 (5) | 8 (14) | - |  |
| **Did it seem like the pain of the patient was under control?** (%yes) | 42 (71) | 49 (84) | 25 (89) | 0.072 |
| **Did the patient die peacefully?** (%yes) | 51 (86) | 50 (86) | 26 (93) | 0.909 |
| End-of-life care and bereavement care for the relatives |  |  |  |  |
| **Where you contacted on time so you could be there when the patient died?** |  |  |  | 0.139 |
| *Yes, I was contacted on time or was already with the patient* | 57 (97) | 50 (86) | 25 (89) |  |
| **Did you and other relatives received sufficient support at the moment of death?** |  |  |  | 0.585 |
| *Very much* | 46 (78) | 43 (74) | 22 (79) |  |
| *A little bit* | 10 (17) | 10 (17) | 2 (7) |  |
| *Not at all* | 2 (3) | 4 (7) | 2 (7) |  |
| *Unknown or missing* | 1 (2) | 1 (2) | 2 (7) |  |
| **Did you receive enough space to properly say goodbye?** (%yes) | 57 (97) | 54 (93) | 26 (93) | 0.549 |
| **Did care professionals treat you tactfully after the patient died?** (%yes) | 57 (97) | 51 (88) | 27 (96) | 0.246 |

^a^P-values of <0.01 were considered statistically significant.
